# Association of rs699947 (−2578 C/A) and rs2010963 (−634 G/C) Single Nucleotide Polymorphisms of the *VEGF* Gene, VEGF-A and Leptin Serum Level, and Cardiovascular Risk in Patients with Excess Body Mass: A Case–Control Study

**DOI:** 10.3390/jcm9020469

**Published:** 2020-02-08

**Authors:** Damian Skrypnik, Adrianna Mostowska, Paweł Piotr Jagodziński, Paweł Bogdański

**Affiliations:** 1 Department of Treatment of Obesity, Metabolic Disorders, and Clinical Dietetics, Poznań University of Medical Sciences, Szamarzewskiego St. 82/84, 60-569 Poznań, Poland; pawelbogdanski73@gmail.com; 2 Department of Biochemistry and Molecular Biology, Poznań University of Medical Sciences, Święcickiego St. 6, 60-781 Poznań, Poland; amostowska@wp.pl (A.M.); pjagodzi@ump.edu.pl (P.P.J.)

**Keywords:** vascular endothelial growth factor, leptin, obesity, VEGF gene polymorphism, cardiovascular risk

## Abstract

Background: Two single nucleotide polymorphisms (SNPs) of the *VEGF* gene, rs699947 and rs2010963, are responsible for differentiated gene expression. A mutual dependence between VEGF and leptin serum level has been observed. This study investigated the associations between the rs699947 and rs2010963 SNPs of *VEGF* gene, VEGF-A, and leptin serum concentrations, and cardiometabolic risk of body mass excess. Methods: In this case–control study, 212 subjects with excess body mass and 145 normal-weight controls gave blood samples and underwent anthropometric and pulse wave analysis. Genotyping of *VEGF* gene was carried out to analyze the rs699947 (−2578 C/A) and rs2010963 (−634 G/C) SNPs. (ClinicalTrials.gov ID: NCT04077554). Results: This study showed a significant positive correlation between serum levels of VEGF-A and leptin in individuals with excess body mass possessing the CC genotype of the rs699947 variant of the *VEGF* gene. It has been registered that an increase in VEGF-A serum level correlates with an increase in arterial stiffness in excess body mass patients harboring AA genotype of the rs699947 (−2578 C/A) variant of the *VEGF* gene. No differences in VEGF-A and leptin serum concentrations were noted between particular genotypes. Conclusions: The CC genotype of the rs699947 variant of the *VEGF* gene promotes a positive interdependency between leptin and VEGF-A serum levels in subjects with excess body mass.

## 1. Introduction

Over 500 million people worldwide presently suffer from obesity, and 50% of the global population has excess body mass [1]. In Poland, 64.1% of men and 46.7% of women are overweight [2]. Excess body mass is a direct causative factor of endothelial dysfunction and its life-threatening consequences, such as atherosclerosis, ischemic heart disease (IHD), myocardial infarction, and stroke [3,4,5].

Endothelial cells synthesize vascular endothelial growth factor (VEGF)—a mitogen responsible for angiogenesis and stimulation of endothelial cells proliferation [6,7,8]. The *VEGF* gene is located on chromosome 6p21.3 [9]. Increased expression of the *VEGF* gene has been registered in human coronary arteries involved in the atherosclerotic process [10]. Blood concentration of VEGF increases with the exacerbation of atherosclerosis [11]. However, the role of VEGF in endothelial and blood vessels dysfunction has not yet been fully clarified. Potentiated accretion of atherosclerotic plaque after VEGF supply has been observed in an animal model [12]. On the other hand, injection of VEGF into damaged arteries has been shown to lead to intensified reendothelization, resulting in diminished arterial stiffness and reduced wall thrombus [13,14]. Studies of VEGF function, expression, and regulation are thus crucial for the full understanding of endothelial dysfunction and for accurate stratification of cardiovascular risk, especially in states that predispose to atherosclerosis, such as excess body mass [15].

A range of single nucleotide polymorphisms (SNPs) of the *VEGF* gene have been described. One SNP in the −634 position and one in the −2578 position in the promoter region are thought to be responsible for differentiated gene expression [16]. These SNPs thus have functional significance. The −634 G and −2578 A alleles are associated with decreased VEGF synthesis in peripheral blood mononuclear cells stimulated with lipopolysaccharide [17,18,19]. Serum concentration of VEGF is decreased in patients homozygous for both −634 G and −2578 A alleles [19,20,21]. Studies to date have also demonstrated that the quantity of atheromatously stenosed coronary vessels correlates positively with the AA genotype of the *VEGF* gene in the −2578 position and negatively with the CC genotype of the *VEGF* gene in the −2578 position. This correlation shows the extremely high clinical importance of VEGF polymorphism for cardiovascular risk [16].

Leptin (LEP) is a hormone the vast majority of which is produced by adipocytes. Obese patients, due to their increased adipose tissue mass and the occurrence of leptin resistance, present high blood LEP concentration [22]. Elevated serum LEP level disturbs endothelial function by increasing reactive oxygen species (ROS) generation and decreasing nitric oxide (NO) synthesis [23]. Thus LEP, like VEGF, is responsible for normal vascular function. In vitro studies have revealed that LEP intensifies the synthesis of VEGF [24,25,26]. The molecular mechanisms of this relation have not yet been investigated. Moreover, there have been no studies of this topic using humans with excess body mass. Thus, our study is the first to attempt to clarify the link between VEGF and leptin serum contents, and *VEGF* gene polymorphism, under conditions of increased adiposity. 

The aim of our study was to examine the associations between the rs699947 (−2578 C/A) and rs2010963 (−634 G/C) single nucleotide polymorphisms of the *VEGF* gene and VEGF-A blood concentration and leptin serum content in Polish patients with excess body mass. We also investigated the connection between *VEGF* gene polymorphism and selected biochemical, functional, and anthropometric factors of cardiometabolic risk and endothelial dysfunction. The novelty of our study arises from the molecular investigatory approach to the influence of leptin on VEGF synthesis in a human excess body mass model, previously not reported, and the analysis of the results in the light of the components of risk of cardiovascular and endothelial dysfunction.

## 2. Experimental Section

### 2.1. Study Design

The study was designed as a cross-sectional case–control study. STROBE (STrengthening the Reporting of OBservational studies in Epidemiology) guidelines were employed. The study protocol was approved by the Ethics Committee at Poznań University of Medical Sciences (approval numbers 359/15 and 1309/18) and fulfilled the requirements of the Declaration of Helsinki (1975 revision with amendments). The study was registered on ClinicalTrials.gov under the ID NCT04077554. The study protocol can be accessed at https://clinicaltrials.gov/ct2/show/NCT04077554. 

The study was performed between March 2016 and September 2017. After screening and enrolling, all patients fulfilling all the inclusion criteria and none of the exclusion criteria were placed in either study group A with excess body mass (BMI ≥ 25 kg/m^2^; *n* = 265) or control group B without excess body mass (BMI < 25 kg/m^2^; *n* = 158). Subsequently, anthropometric measurements, blood pressure and heart rate measurements, pulse wave analysis, and blood sample collection were performed on all patients. Subjects were excluded from the analysis of they did not have all necessary measurements made, or if blood samples were not collected from them. The study procedures and data collection and analysis were performed at the Department of Treatment of Obesity, Metabolic Disorders and Clinical Dietetics, Poznań University of Medical Sciences. 

### 2.2. Study Participants

Written informed consent was obtained from each patient. Subjects were all inhabitants of five randomly chosen cities in Western Poland. The inclusion criteria were: informed consent in writing, age ≥ 18 years, body mass index (BMI) ≥ 25 kg/m^2^ (study group, group A) or BMI < 25 kg/m^2^ (control group, group B), and stable body mass (±1 kg) during the month prior enrollment. The exclusion criteria were: age < 18 years, secondary form of obesity, clinically significant inflammatory state, infection in the month prior to enrollment, pregnancy or lactation, or any state that could prevent the efficacy of the study or limit the credibility of the results. Patients’ sex and age were self-reported. Obesity and overweight are been defined in line with the World Health Organization (WHO) criteria as BMI ≥ 30.0 kg/m^2^ and BMI 25.0–29.9 kg/m^2^, respectively; similarly, subjects with BMI < 25 kg/m^2^ were considered not to have body mass excess [27]. 

### 2.3. Anthropometric Measurements

Anthropometric measurements were performed under metabolic laboratory conditions, with patients wearing light clothes and no shoes, in the morning, after overnight fasting and sleep, what enabled us to minimize potential measurement bias. Body mass was measured to the nearest 0.1 kg using electronic scales (WPT 100/200 OW; Radwag, Radom, Poland) and height to the nearest 1 cm using a manual stadiometer (WPT 100/200 OW; Radwag, Radom, Poland). The body mass index (BMI) was calculated by dividing the body mass [kg] by the height [m] squared. Waist circumference (WC) was measured at the middle point between the iliac crest and the lowest rib and neck circumference (NC) was measured from the level just underneath the laryngeal prominence perpendicular to the long axis of the neck with the use of tape.

### 2.4. Circulatory Parameters

Circulatory parameters were determined in the morning, after overnight fasting and sleep, under metabolic laboratory conditions, after 15 min rest in sitting position in silence, at room temperature, without previous coffee consumption on the day of measurement. These measurement conditions allowed us to minimize potential measurement bias. Resting blood pressure (BP) was measured from the arm, with the patient in a sitting position, back and upper limb supported. A digital electronic tensiometer (model 705IT, Omron Corporation, Kyoto, Japan) and small, regular, or large adult cuffs, depending on the patient’s arm circumference, were used. Resting heart rate (HR) was determined under the same conditions. Pulse wave analysis (PWA) was performed with a noninvasive method (photoplethysmography) using a Pulse Trace PCA2 (CareFusion Corporation, San Diego, CA, USA) device. The pulse waveform has been obtained from the patient’s finger. The following pulse waveform parameters were calculated: peak-to-peak time (PPT), which is the time from systolic inflection point or first peak to the second peak or inflection point (this depends on the speed of pulse wave propagation); the stiffness index (SI), which is the subject’s height divided by PPT (this describes the properties of the large arteries); and the reflection index (RI), which is the height of the second peak or inflection point divided by the height of the first peak (this allows quantitative assessment of the impact of wave reflection). Increased arterial stiffness is documented by increased values of SI and RI and by low values of PPT [28].

### 2.5. Blood Sample Collection

Blood samples were collected in the morning from patients who had slept and fasted all night, after lying supine half an hour in silence, at room temperature, with no previous coffee consumption on the day of blood collection. Ulnar vein blood samples were collected into ethylenediaminetetraacetic acid (EDTA) tubes to obtain whole blood and into serum separated tubes. After preparation, the blood samples were analyzed or frozen immediately after collection and stored at −80 °C, allowing us to minimize potential bias in analysis.

### 2.6. Biochemical Analysis

Serum concentrations of VEGF-A and leptin were determined using enzyme-linked immunosorbent assay (ELISA). Commercial kits (Elabscience, Houston, TX, USA for VEGF-A and leptin) and Synergy spectrometer (BioTek Instruments, Winooski, VT, USA) were used. Serum concentrations of glucose (GLU), total cholesterol (TCH), high-density lipoprotein (HDL), triglycerides (TG), C-reactive protein (CRP), alanine transaminase (ALAT), aspartate transaminase (ASPAT), creatinine (Creat), and uric acid (UA) were measured by routine enzymatic methods in a commercial laboratory. The low-density lipoprotein (LDL) serum concentration was calculated using the Friedewald formula [29] and the glomerular filtration rate (eGFR) was determined using the Cockcroft–Gault formula [30].

### 2.7. Genotyping

Genomic deoxyribonucleic acid (DNA) was isolated from peripheral blood lymphocytes using the salt-out extraction procedure. Genotyping of the VEGF nucleotide variants was carried out by high-resolution melting curve analysis (HRM) on the LightCycler 96 system (Roche Diagnostics, Mannheim, Germany) with the use of 5 × HOT FIREPol EvaGreen HRM Mix (Solis BioDyne, Tartu, Estonia). Two *VEGF* variants, rs699947 and rs2010963, were analyzed. For the rs2010963 variant, to distinguish between CC and GG genotype, all homozygous samples were spiked with a wild-type DNA and reanalyzed. As a quality control measure, approximately 10% of the randomly selected samples were genotyped in duplicate to check for concordance. Samples that failed the genotyping were excluded from further statistical analysis. Primer sequences and HRM conditions are presented in Table 1.

### 2.8. Statistical Analysis

Statistical analysis was performed using Dell Statistica version 13 (2016, Dell, Tulsa, OK, USA). Continuous variables were summarized using mean, standard deviation, median, and quartile range; categorical variables were summarized using frequency and percentages. All quantitative results were first verified by the Shapiro–Wilk normality test. The results for the study group were compared with the results for the control group using the paired *t*-test (when the distribution of variables was consistent with the normal distribution) or the Mann–Whitney *U*-test (when the Shapiro–Wilk test confirmed a lack of normality). The Kruskal–Wallis test was use to compare VEGF-A and leptin serum concentrations between particular genotypes of *VEGF* gene variants. 

The sample size was determined according to VEGF-A serum concentrations. It was calculated that a sample size of at least 122 subjects in group A and B separately would yield at least 80% power of detecting the significant difference. Missing data was not included into the statistical analyses.

The chi-square test was used to compare the genotype frequencies of the −2578 C/A and −634 G/C variants of each group for all subjects, and then separately for women and men. Two subgroups of serum concentration of VEGF-A and leptin were also determined: below and above the median. The chi-test was also used to determine the dependency between the subgroups and genotypes.

Correlations between the serum level of VEGF-A or leptin and the clinical data were determined using Spearman’s correlation coefficient for groups and genotypes. Depending on the R-value correlations were considered weak (R 0.0–0.3), moderate (R 0.3–0.5), strong (R 0.5–0.7), or very strong (R > 0.7) [31]. The results were accepted as significant at *p* < 0.05.

## 3. Results

A random group of 1128 subjects were screened and 423 fulfilled the inclusion criteria and presented no exclusion criteria. In addition, 66 subjects were excluded because of lack of all required measurements. Thus, the final study population consisted of 212 patients in group A (BMI ≥ 25 kg/m^2^) and 145 patients in group B (BMI < 25 kg/m^2^). Mean age of the patients was 60.28 years in group A and 42.53 years in group B. Due to limited availability of the PCA2 Pulse Trace, PWA was performed in part of the study population (172 subjects in group A and 68 subjects in group B). To avoid potential bias, PWA was performed in randomly chosen subjects, which made the results highly credible. A flow diagram of the study is presented as Figure S1.

The anthropometric parameters of body mass, BMI, WC, and NC were higher in group A than in group B. On the other hand, the control group was taller than group A. The functional parameters of the cardiovascular system— SBP: systolic blood pressure, DBP: diastolic blood pressure, and SI: stiffness index—presented higher values in patients with excess body mass than in the controls, who, on the other hand, had higher PPT. The groups did not differ in HR or RI. Group A demonstrated higher serum concentration of the biochemical parameters leptin, GLU, TCH, TG, Creat, ALT, AST, CRP, and UA than did group B, while the eGFR value and serum concentrations of VEGF-A and HDL were higher in the control group than in subjects with excess body mass. A comparison of anthropometric, functional, and biochemical parameters between groups A and B is given in Table 2.

Statistical analysis of the entire study population together, and of women and men separately, revealed no differences between groups A and B in the distribution of the particular genotypes of the rs699947 and rs2010963 variants of the *VEGF* gene. In the entire sample, the minor allele frequencies (MAF) for the tested nucleotide variants were 0.483 (allele C) and 0.248 (allele C), respectively. The distribution of the rs699947 and rs2010963 genotype variants of the *VEGF* gene are presented in Table 3 and Table 4, respectively.

Comparison of the particular genotypes of rs699947 and rs2010963 VEGF variants revealed no differences in VEGF-A and leptin serum concentrations between genotypes in the entire study population and in groups A and B separately. Table 5 presents a comparison of the VEGF-A and leptin serum concentrations between the particular genotypes of the rs699947 and rs2010963 variants of the *VEGF* gene.

The analysis revealed no differences in the distribution of the rs699947 and rs2010963 SNPs of the *VEGF* gene between the subgroups with serum concentrations of VEGF-A and leptin below and above the median in the entire study population and in groups A and B separately. The distributions of rs699947 and rs2010963 in relation to the serum concentration of VEGF-A and leptin below and above the median are presented inTable 6 and Table 7 respectively.

Our study found a number of significant correlations between VEGF-A and leptin serum concentrations and the examined parameters. In individuals harboring the GG genotype of the rs2010963 (−634 G/C) SNP, the serum VEGF-A concentration correlated negatively with eGFR in group A. In patients harboring the GG genotype of the rs2010963 (−634 G/C) SNP, the serum leptin level correlated negatively with height and with NC and positively with HR, CRP serum level, and HDL serum level in group A. It also correlated negatively with height and NC, as well as negatively with height in the entire study population. In individuals harboring the CG genotype of rs2010963 (−634 G/C), the serum VEGF-A concentration correlated negatively with RI in group B. In subjects with the CG genotype of rs2010963 (−634 G/C), the serum leptin level correlated positively with PPT in group B, and negatively with height, RI, and ALT serum levels in group A; it also correlated negatively with SI in group in group B, and negatively with height in the entire study population. In individuals harboring the CC genotype of the rs2010963 (−634 G/C) variant, the serum VEGF-A concentration correlated positively with HR, DBP, and HDL serum level, and negatively with eGFR in group A, as well as positively with HR in the entire study population. 

In individuals harboring the AA genotype of rs699947 (−2578 C/A), the serum VEGF-A concentration correlated positively with glucose serum level and SI, and negatively with PPT in group A. It also correlated positively with glucose serum level and negatively with TCH and LDL serum concentrations in group B, as well as positively with glucose serum concentration in the entire study population. In individuals harboring the CC genotype of rs699947 (−2578 C/A), the serum VEGF-A concentration correlated positively with leptin and HDL serum concentrations, as well as negatively with NC and ALT serum content in group A. It also correlated positively with HR in group B, positively with PPT, and negatively with SI in the entire study population. In subjects with the AA genotype of rs699947 (−2578 C/A), the serum leptin concentration correlated positively with HR in group A, group B and in the entire study population. In individuals with the AC genotype of rs699947 (−2578 C/A), the serum leptin concentration correlated positively with CRP serum level and PPT in group A, and negatively with height in group A, group B, and the entire study population. It also correlated negatively with NC in groups A and B, and negatively with SI and RI in group A. In individuals harboring the CC genotype of rs699947 (−2578 C/A), the serum leptin concentration correlated positively with VEGF-A and HDL serum levels and negatively with ALT serum concentration in group A. It also correlated negatively with the TCH serum level in group B. Significant correlations of VEGF-A and leptin serum contents with other parameters were found in relation to the individual genotypes of the rs2010963 (−634 G/C) and rs699947 (−2578 C/A) variants of the *VEGF* gene and these are shown in Table 8 and Table 9, respectively.

## 4. Discussion

In this study, we examined the connection between *VEGF* gene polymorphism, VEGF-A, and leptin blood concentration and a range of cardiovascular risk parameters and endothelial function parameters in overweight and obese patients in a Polish population. We have shown for the first time a significant positive correlation between serum concentrations of VEGF-A and leptin in individuals with the CC genotype of the rs699947 (−2578 C/A) variant of *VEGF* gene in a population with excess body mass.

Comparing groups A and B for anthropometric, functional, and biochemical parameters, it was clear that those patients with BMI ≥ 25 kg/m^2^ had significantly higher cardiovascular risk levels than the control group. This higher cardiovascular risk can be seen in the first instance in the higher values of BMI, WC [32], SBP, and DBP [33]. The higher serum concentrations of TCH [34], TG [32], Creat [32], ALT [35], and UA [36], as well as the lower serum levels of HDL [32] and eGFR [32] in this group, also point to an unfavorable cardiometabolic profile. Finally, group A presented significant endothelial dysfunction, shown mainly by the elevated level of SI and the lower level of PPT [37,38], but also by higher serum concentration of UA and TCH [39]. 

Recent studies have demonstrated that VEGF-A has a potential role in adipocyte function and in the regulation of energy balance. Elias et al. noted that high expression of the *VEGF* gene prevents diet-induced obesity and increases insulin sensitivity. Moreover, increased expression of VEGF enhances expression of peroxisome proliferator-activated receptor gamma coactivator 1-alpha (PGC-1α) and of uncoupling protein 1 (UCP-1), intensifies thermogenesis and energy expenditure in brown adipose tissue (BAT), and promotes a “BAT-like” phenotype in white adipose tissue (WAT) [40]. Our study found lower VEGF-A and higher CRP serum concentrations in patients with excess body mass, accompanied by markers of endothelial dysfunction, such as higher SI and lower PPT. Furthermore, the VEGF-A serum level in group B correlated negatively with RI in the CG genotype of the rs2010963 (−634 G/C) variant of the *VEGF* gene. On the other hand, in overweight and obese patients with the AA genotype of the rs699947 (−2578 C/A) SNP, serum VEGF-A concentration correlated positively with SI and negatively with PPT. Our study has thus provided a clinical approach to the results of Elias et al. and documented the anti-obesity and anti-inflammatory role of serum VEGF-A, as well as its ability to protect endothelium function. Based on our study, it can be hypothesized that these properties of VEGF-A are dependent on nutrition status and *VEGF* gene polymorphism.

Allele distribution of rs699947 and rs2010963 SNPs similar to this registered in our study is reported for the European (non-Finnish) population from the Genome-Aggregation Database (gnomAD genomes, http://gnomad.broadinstitute.org/). In this large population, the MAF values for VEGF variants are 0.486 and 0.291, respectively. When we analyzed the distribution of the genotypes of rs699947 (−2578 C/A) and rs2010963 (−634 G/C), we found no differences between overweight and normal-weight subjects. This suggests that these two polymorphisms had no effect on the patients’ nutritional status. Also, we found no significant differences between the VEGF-A serum levels of the rs699947 (−2578 C/A) and rs2010963 (−634 G/C) variant genotypes. Similarly, no difference was seen in the distribution of the rs699947 and rs2010963 genotypes in subjects with VEGF-A serum content below and above the median. This suggests that VEGF-A blood concentration is not dependent on the *VEGF* gene’s rs699947 (−2578 C/A) and rs2010963 (−634 G/C) polymorphisms, in disagreement with the results of Howell et al. [16].

In relation to the rs2010963 (−634 G/C) variant, we found a range of significant correlations between the serum VEGF-A level and functional and biochemical parameters: positive correlations with HR, DBP, and HDL serum level in the CC genotype in obese and overweight patients, and negative correlations with eGFR, also in group A, in the GG and CC genotypes. HR, DBP, serum HDL, and eGFR have been demonstrated to be markers of cardiovascular risk [41,42,43,44]. This suggests that, particularly in patients with the CC genotype, the synthesis of VEGF-A, and consequently the process of angiogenesis and endothelial dysfunction management, is susceptible to overall cardiometabolic status. The discrepancy in terms of the correlation with HR and DBP vs. HDL and eGFR levels shows that the molecular and biochemical basis of the correlation requires further investigation. Interestingly, Iaresko et al. [45] have demonstrated that the GG genotype of the rs2010963 (−634 G/C) variant of the *VEGF* gene is common in obese women with arterial hypertension in premenopause. Moreover, Iaresko et al. have shown a higher level of VEGF in females with the GG genotype of the rs2010963 (−634 G/C) variant than in subjects with CC and GC genotypes. Considering these results and our own, we can state that the GG and CC genotypes are significantly connected with patients’ cardiovascular risk, with a particular emphasis on the role of the GG genotype in the development of hypertension in obesity.

Howell et al. showed that, in relation to the rs699947 (−2578 C/A) variation, the number of atheromatously stenosed coronary vessels correlates positively with AA genotype and negatively with CC genotype [16]. The results of our study confirmed to some extent the observations of Howell et al. by demonstrating that VEGF-A serum concentration correlates positively with SI and negatively with PPT in patients with excess body mass with AA genotype. Conversely, VEGF-A serum concentration correlates negatively with SI and positively with PPT in the entire study population with CC genotype. Our findings allow us to hypothesize that the association of the rs699947 (−2578 C/A) variant of the *VEGF* gene with coronary atheromatosis demonstrated by Howell et al. may be dependent on the patient’s nutritional status through some not well understood mechanism. The correlations found in our study demonstrate that blood lipid status may play a significant role in a subtle cross-talk between the polymorphism of the rs699947 (−2578 C/A) variant of the *VEGF* gene and risk of atheromatosis: we found that, in normal weight subjects with the AA polymorphism, serum VEGF-A correlated negatively with serum TCH and LDL. In patients with excess body mass and the CC polymorphism, serum VEGF-A correlated positively with serum HDL.

We have shown that, in patients with excess body mass, serum VEGF-A and leptin concentrations are positively correlated. Such a correlation was seen only in individuals carrying the CC genotype of the rs699947 (−2578 C/A) variant of the *VEGF* gene and was not noted in subjects with BMI < 25 kg/m^2^. No such result has previously been observed in a human model. Lu et al. [46] reported a 60% reduction in leptin gene expression under conditions of repression of *VEGF* gene expression in a mouse model. This finding may constitute a molecular rationale for our results, which on the other hand provided a clinical manifestation of the observation of Lu et al. Based on these outcomes, we hypothesize that *VEGF* and leptin gene expression is coregulated by a mechanism probably dependent on *VEGF* gene polymorphism and the subject’s nutritional status. Lu et al. documented upregulation of BAT specific genes, such as UCP-1, GATA-1, CIDEA, PRDM16, and BMP-7, in VEGF-expression-repressed mice.

A range of in vitro studies have also revealed that leptin intensifies the production of VEGF. In these studies the significant role of nuclear factor kappa-light-chain-enhancer of activated B cells (NFkappaB), hypoxia-inducible factor 1alpha (HIF-1alpha) [24]; VEGFR2 (VEGF receptor 2), Notch, and interleukin 1 (IL1) genes, Notch, IL1, and leptin crosstalk outcome (NILCO) [25]; focal adhesion kinase (FAK), phosphatidylinositol 3-kinase (PI3K), and protein kinase B (Akt) [26] has been documented. Our findings correspond with these previous observations, suggesting that the effect of leptin on VEGF synthesis is significant in subjects with the CC genotype of the rs699947 (−2578 C/A) variant of the *VEGF* gene and who possess an excess of body mass. 

Our study showed that leptin serum content is independent of the *VEGF* gene’s rs699947 (−2578 C/A) and rs2010963 (−634 G/C) polymorphisms. However, we noted moderate correlations of leptin serum concentration in patients with the CG genotype of the rs2010963 (−634 G/C) variant: in normal-weight subjects, the correlation was negative with SI and positive with PPT and in patients with excess body mass, the correlation was negative with RI. The same correlations were found in overweight and obese subjects with the AC genotype of rs699947 (−2578 C/A). Jamroz-Wiśniewska et al. have demonstrated that the reduction in NO synthesis caused by leptin is compensated for by the upregulation of endothelium-derived hyperpolarizing factors (EDHF), especially under conditions of chronic hyperleptinemia and body mass excess. EDHF-induced leptin-derived vasorelaxation is partially mediated by hydrogen sulfide (H_2_S) [47]. 

### 4.1. Study Limitations

The most significant limitation of this trial is its territorial restriction to Western Poland, which mainly only allows the results to be related to this population. Moreover, a relatively small number of subjects were included. This was mainly due to the strict inclusion and exclusion criteria, which allowed us to select a study group that was not encumbered by states that might significantly limit the quality and credibility of the results. Further, the allele distribution of the rs699947 and rs2010963 SNPs noted in our study is similar to that reported for the European (non-Finnish) population from the Genome-Aggregation Database. Also, PWA was performed only in part of the studied population (172 subjects in group A and 68 subjects in group B) due to the limited availability of equipment. However, the patients for whom PWA analysis was performed were chosen randomly, meaning the results are highly credibility.

### 4.2. Study Strong Points

The greatest strong point of this trial is its molecular approach to the question of the mutual influence of leptin and VEGF: we analyzed two SNPs of the *VEGF* gene, one at the −634 position and one at the −2578 position of the promoter region, which are stated to be responsible for differentiated gene expression [16]. Moreover, the effect of leptin on VEGF has so far been investigated only in vitro and animal studies. Our trial revealed the codependency of leptin and VEGF in a human model of excess body mass and its relation to the SNPs of the *VEGF* gene and this constitutes the pioneering nature of our study. We also succeeded in analyzing a broad range of anthropometric, functional and biochemical parameters of cardiovascular risk, showing for the first time its possible relation to SNPs of the *VEGF* gene, especially in terms of arterial stiffness indices.

## 5. Conclusions

Our results did not succeed in showing a significant influence of the rs2010963 (−634 G/C) and rs699947 (−2578 C/A) variants of the *VEGF* gene on leptin or VEGF-A serum concentrations in patients with excess body mass. However, based on our results, we can conclude that the CC genotype of the rs699947 (−2578 C/A) variant of the *VEGF* gene promotes the mutual positive influence of leptin and VEGF-A serum levels in overweight and obese patients. We have additionally shown that the mutual influence of leptin and VEGF-A serum levels and cardiovascular risk parameters—especially arterial stiffness parameters—is dependent on the SNPs of the *VEGF* gene examined here and on patients’ nutritional status. In patients with excess body mass and AA genotype of the rs699947 (−2578 C/A) variant of the *VEGF* gene, an increase in VEGF-A serum level increases arterial stiffness. Conversely, in members of the general population with the CC genotype, high VEGF-A serum concentration promotes decreased arterial stiffness. In clinical practice, this conclusion suggests the inclusion of simultaneous serum VEGF-A level assessment and analysis of the rs699947 (−2578 C/A) variant of the *VEGF* gene into the overall process of estimating the cardiovascular risk of patients with excess body mass, in order to increase the precision of risk profile determination. Further investigation into the effects of SNPs of the *VEGF* gene on cardiovascular risk in obesity is needed to draw a more precise conclusion.

## Figures and Tables

**Table 1:** Primers and high-resolution melting curve analysis (HRM) conditions for genotyping of vascular endothelial growth factor (*VEGF)* gene variants.

rs No.	Chromosome Location ^a^	Alleles	Primers for PCR Amplification (5′–3′)	PCR Product Length (bp)	Annealing Temp. (°C)	Melt. Temp. Range (°C)
**rs699947**	chr6:43768652	A/C	F: ATTCTCAGTCCATGCCTCCA	79	52	80–95
			R: CAGTCTGATTATCCACCCAGA			
**rs2010963**	chr6:43770613	C/G	F: GTGGATTTTGGAAACCAGCA	142	52	80–95
			R: AAAGCAGGTCACTCACTTTGC	analysis without and with spiking DNA		

^a^ GRCh38/hg38; HRM: high-resolution melting curve analysis; PCR: polymerase chain reaction; VEGF: vascular endothelial growth factor.

**Table 2:** Comparison of analyzed parameters between group A and B.

Parameter	Group A		Group B		*p*-Value
	n	Mean ± SD	n	Mean ± SD	
Body mass [kg]	212	88.06 ± 15.40	145	62.80 ± 8.77	**<0.0001**
Height [cm]	212	164.14 ± 12.56	145	167.25 ± 8.20	**<0.05**
BMI [kg/m^2^]	212	32.08 ± 4.15	145	22.28 ± 1.84	**<0.0001**
WC [cm]	212	105.22 ± 12.06	145	79.81 ± 9.82	**<0.0001**
NC [cm]	212	38.70 ± 5.13	145	33.57 ± 2.76	**<0.0001**
SI	172	8.61 ± 4.87	68	6.66 ± 2.12	**<0.0001**
PPT [ms]	172	225.33 ± 69.34	68	262.09 ± 65.85	**<0.0001**
RI [%]	172	54.60 ± 20.33	68	53.80 ± 30.13	NS *
SBP [mmHg]	212	142.12 ± 18.86	144	121.83 ± 16.89	**<0.0001**
DBP [mmHg]	212	84.38 ± 12.39	144	75.99 ± 8.29	**<0.0001**
HR [BPM]	172	76.56 ± 13.33	140	78.16 ± 13.22	NS *
VEGF-A [pg/mL]	212	218.94 ± 197.80	145	322.40 ± 314.94	**<0.05**
Leptin [ng/mL]	212	26.55 ± 21.35	145	14.03 ± 12.66	**<0.0001**
GLU [mg/dL]	211	101.30 ± 33.10	145	88.66 ± 16.20	**<0.0001**
TCH [mg/dL]	208	201.58 ± 42.79	141	191.29 ± 35.72	**<0.05**
HDL [mg/dL]	208	58.44 ± 14.51	142	70.42 ± 17.41	**<0.0001**
LDL [mg/dL]	199	111.95 ± 50.20	141	104.26 ± 47.75	NS *
TG [mg/dL]	208	185.68 ± 106.50	142	112.61 ± 76.45	**<0.0001**
Creat [mg/dL]	208	0.80 ± 2.70	142	0.71 ± 0.14	**<0.0001**
eGFR [ml/min/m^2^]	204	59.49 ± 1.33	141	60.00 ± 0.03	**<0.05**
AST [U/L]	208	29.41 ± 11.58	142	25.04 ± 6.62	**<0.0001**
ALT [U/L]	208	36.49 ± 21.55	142	22.97 ± 10.05	**<0.0001**
UA [g/dl]	208	5.77 ± 1.33	142	4.28 ± 0.99	**<0.0001**
CRP [mg/l]	208	5.05 ± 2.87	141	4.17 ± 1.11	**<0.001**

ALT: alanine transaminase; AST: aspartate transaminase; BMI: body mass index; Creat: creatinine; CRP: C-reactive protein; DBP: diastolic blood pressure; eGFR: estimated glomerular filtration rate; GLU: glucose; HDL: high density lipoprotein; HR: heart rate; LDL: low density lipoprotein; NC: neck circumference; NS-not significant; PPT: peak-to-peak time; RI: reflection index; SBP: systolic blood pressure; SD: standard deviation; SI: stiffness index; TCH: total cholesterol; TG: triglycerides; UA: uric acid; VEGF-A: vascular endothelial growth factor A; WC: waist circumference; * normal distribution.

**Table 3:** Distribution of genotypes of rs699947 (2578 C/A) variant of *VEGF* gene.

rs699947 (−2578 C/A) Variant	Total		Women		Men	
	Group A (*n* = 212)	Group B (*n* = 145)	Group A (*n* = 140)	Group B (*n* = 113)	Group A (*n* = 72)	Group B (*n* = 32)
AA	50	44	36	34	14	10
% of column	24%	30.5%	25.5%	30%	19%	31%
AC	111	70	68	52	43	18
% of column	52%	48%	49%	46%	60%	56%
CC	51	31	36	27	15	4
% of column	24%	21.5%	25.5%	24%	21%	13%
Chi^2^; *p*-value	Chi^2^ = 2.05; *p* = 0.3595		Chi^2^ = 0.60; *p* = 0.7402		Chi^2^ = 2.23; *p* = 0.3286	

**Table 4:** Distribution of genotypes of rs2010963 (634 G/C) variant of *VEGF* gene.

rs2010963 (−634 G/C) Variant	Total		Women		Men	
	Group A (*n* = 212)	Group B (*n* = 145)	Group A (*n* = 140)	Group B (*n* = 113)	Group A (*n* = 72)	Group B (*n* = 32)
GG	118	88	79	70	39	18
% of column	56%	61%	57%	62%	54%	56%
CG	77	48	48	34	29	14
% of column	36%	33%	34%	30%	40%	44%
CC	17	9	13	9	4	0
% of column	8%	6%	9%	8%	6%	0%
Chi^2^; *p*-value	Chi^2^ = 1.02; *p* = 0.6004		Chi^2^ = 0.78; *p* = 0.6741		Chi^2^ = 1.86; *p* = 0.3946	

**Table 5:** Comparison of VEGF-A and leptin serum concentrations between particular genotypes of *VEGF* gene variants.

Serum Concentration/VEGF Gene Variant		Total			Group A			Group B		
	Genotype	*n*	Mean ± SD	*p* K-W	*n*	Mean ± SD	*p* K-W	*n*	Mean ± SD	*p* K-W
VEGF-A [pg/mL]/rs699947 (−2578 C/A)	AA	94	253.02 ± 227.07	0.397	50	175.22 ± 95.71	0.2529	44	341.43 ± 293.32	0.6066
	AC	181	266.45 ± 290.53		111	232.38 ± 241.17		70	320.48 ± 350.21	
	CC	82	257.97 ± 206.65		51	232.58 ± 160.20		31	299.74 ± 263.86	
VEGF-A [pg/mL]/rs2010963 (−634 G/C)	GG	206	259.27 ± 242.70	0.8416	118	219.19 ± 204.95	0.6815	88	313.01 ± 277.86	0.9972
	CG	125	267.43 ± 292.37		77	215.54 ± 195.55		48	350.68 ± 390.13	
	CC	26	243.32 ± 175.67		17	232.66 ± 164.02*		8	263.45 ± 204.78*	
Leptin [ng/mL]/rs699947 (−2578 C/A)	AA	94	21.92 ± 22.58	0.4918	50	29.31 ± 27.13	0.7902	44	13.53 ± 11.36	0.9739
	AC	181	21.31 ± 19.30		111	25.86 ± 21.13		70	14.09 ± 13.17	
	CC	82	21.29 ± 15.03		51	25.34 ± 14.52		31	14.62 ± 13.57	
Leptin [ng/mL]/rs2010963 (−634 G/C)	GG	206	21.54 ± 19.90	0.9769	118	27.78 ± 23.01	0.937	88	13.18 ± 9.87	0.5805
	CG	125	21.73 ± 19.24		77	25.32 ± 20.09		48	15.98 ± 16.41	
	CC	26	19.55 ± 14.91		17	23.52 ± 14.01		9	12.04 ± 14.32	

*p* K-W: Kruskal–Wallis *p*-value; SD: standard deviation; VEGF-A: vascular endothelial growth factor A; * normal distribution.

**Table 6:** Distribution of genotypes of rs699947 and rs2010963 variants of *VEGF* gene in relation to serum concentration of VEGF-A below and above the median.

rs699947 (−2578C/A) Polymorphism	Total		Group A		Group B	
	Below Median (*n* = 177)	Above Median (*n* = 180)	Below Median (*n* = 114)	Above Median (*n* = 98)	Below Median (*n* = 63)	Above Median (*n* = 82)
AA	46	48	29	21	17	27
% of column	26%	26.5%	25.44%	21.43%	26.98%	32.93%
AC	97	84	63	48	34	36
% of column	55%	47%	55.26%	48.98%	53.97%	43.90%
CC	34	48	22	29	12	19
% of column	19%	26.5%	19.30%	29.59%	19.05%	23.17%
Chi^2^; *p*-value	Chi^2^ = 3.34; *p* = 0.1881		Chi^2^ = 3.08; *p* = 0.2146		Chi^2^ = 1.44; *p* = 0.4854	
**rs2010963 (−634 G/C) Polymorphism**	**Below median (*n* = 177)**	**Above median (*n* = 180)**	**Below median (*n* = 114)**	**Above median (*n* = 98)**	**Below median (*n* = 63)**	**Above median (*n* = 82)**
GG	102	104	64	54	38	50
% of column	57.5%	58%	56%	55%	60%	61%
CG	67	58	45	32	22	26
% of column	38%	32%	39.5%	33%	35%	32%
CC	8	18	5	12	3	6
% of column	4.5%	10%	4.5%	12%	5%	7%
Chi^2^; *p*-value	Chi^2^ = 4.49; *p* = 0.1060		Chi^2^ = 4.74; *p* = 0.0933		Chi^2^ = 0.49; *p* = 0.7833	

VEGF-A: vascular endothelial growth factor A.

**Table 7:** Distribution of genotypes of rs699947 and rs2010963 variants of *VEGF* gene in relation to serum concentration of leptin below and above the median.

rs699947 (−2578 C/A) Polymorphism	Total		Group A		Group B	
	Below Median (*n* = 181)	Above Median (*n* = 176)	Below Median (*n* = 83)	Above Median (*n* = 129)	Below Median (*n* = 98)	Above Median (*n* = 47)
AA	52	42	22	28	30	14
% of column	29%	24%	27%	22%	31%	30%
AC	91	90	44	67	47	23
% of column	50%	51%	53%	52%	48%	49%
CC	38	44	17	34	21	10
% of column	21%	25%	20%	26%	21%	21%
Chi^2^; *p*-value	Chi^2^ = 1.44; *p* = 0.4871		Chi^2^ = 1.23; *p* = 0.9932		Chi^2^ = 0.14; *p* = 0.9932	
**rs2010963 (−634 G/C) polymorphism**	**Below median (*n* = 181)**	**Above median (*n* = 176)**	**Below median (*n* = 83)**	**Above median (*n* = 129)**	**Below median (*n* = 98)**	**Above median (*n* = 47)**
GG	108	98	48	70	60	28
% of column	59.5%	56%	58%	54%	61%	60%
CG	61	64	30	47	31	17
% of column	34%	36%	36%	37%	32%	36%
CC	12	14	5	12	7	2
% of column	6.5%	8%	6%	9%	7%	4%
Chi^2^; *p*-value	Chi^2^=0.64; *p* = 0.7257		Chi^2^ = 0.79; *p* = 0.6725		Chi^2^ = 0.63; *p* = 0.7170	

**Table 8:** Significant correlations of VEGF-A and leptin serum concentrations for rs2010963 (−634 G/C) variant of *VEGF* gene. Group A-study group. Group B-control group.

Correlations of VEGF-A Serum Concentration							
Parameter	Genotype	Group	R	Parameter	Genotype	Group	R
eGFR	GG	A	−0.19	DBP	CC	A	0.49
RI	CG	B	−0.48	eGFR	CC	A	−0.52
HR	CC	A + B	0.45	HDL	CC	A + B	0.46
HR	CC	A	0.61	HDL	CC	A	0.59
**Correlations of Leptin Serum Concentration**							
**Parameter**	**Genotype**	**Group**	**R**	**Parameter**	**Genotype**	**Group**	**R**
Height	GG	A + B	−0.33	HDL	GG	A	0.31
Height	GG	A	−0.38	Height	CG	A + B	−0.29
Height	GG	B	−0.22	Height	CG	A	−0.24
NC	GG	A	−0.26	SI	CG	B	−0.46
NC	GG	B	−0.22	PPT	CG	B	0.48
HR	GG	A	0.23	RI	CG	A	−0.38
CRP	GG	A	0.20	ALT	CG	A	−0.31

ALT: alanine transaminase; CRP: C-reactive protein; DBP: diastolic blood pressure; eGFR: estimated glomerular filtration rate; HDL: high density lipoprotein; HR: heart rate; NC: neck circumference; PPT: peak-to-peak time; RI: reflection index; SBP: systolic blood pressure; SI: stiffness index; VEGF-A: vascular endothelial growth factor A.

**Table 9:** Significant correlations of VEGF-A and leptin serum concentrations for rs699947 (−2578 C/A) variant of *VEGF* gene. Group A-study group. Group B-control group.

Correlations of VEGF-A Serum Concentration							
Parameter	Genotype	Group	R	Parameter	Genotype	Group	R
GLU	AA	A + B	0.34	Leptin	CC	A	0.30
GLU	AA	A	0.46	NC	CC	A	−0.31
GLU	AA	B	0.45	SI	CC	A + B	−0.29
SI	AA	A	0.37	PPT	CC	A + B	0.31
PPT	AA	A	−0.36	HR	CC	B	0.40
TCH	AA	B	−0.33	ALT	CC	A	−0.30
LDL	AA	B	−0.33	HDL	CC	A	0.33
**Correlations of Leptin Serum Concentration**							
**Parameter**	**Genotype**	**Group**	**R**	**Parameter**	**Genotype**	**Group**	**R**
HR	AA	A + B	0.32	SI	AC	A	−0.29
HR	AA	A	0.33	PPT	AC	A	0.28
HR	AA	B	0.33	RI	AC	A	−0.42
Height	AC	A + B	−0.42	CRP	AC	A	0.26
Height	AC	A	−0.43	VEGF−A	CC	A	0.30
Height	AC	B	−0.42	ALT	CC	A	−0.33
NC	AC	A	−0.26	TCH	CC	B	−0.42
NC	AC	B	−0.38	HDL	CC	A	0.32

ALT: alanine transaminase; CRP: C-reactive protein; GLU: glucose; HDL: high density lipoprotein; HR: heart rate; LDL: low density lipoprotein; NC: neck circumference; PPT: peak-to-peak time; RI: reflection index; SI: stiffness index; TCH: total cholesterol; VEGF-A: vascular endothelial growth factor A.

## Supplementary Materials

The following are available online at https://www.mdpi.com/2077-0383/9/2/469/s1, Figure S1. Flow diagram of the study.
