# Supplementary material for: Association of rs699947 (−2578 C/A) and rs2010963 (−634 G/C) Single Nucleotide Polymorphisms of the VEGF Gene, VEGF-A and Leptin Serum Level, and Cardiovascular Risk in Patients with Excess Body Mass: A Case–Control Study
Source: J Clin Med. 2020 Feb 8;9(2):469. doi: 10.3390/jcm9020469 (PMC7073834; doi:10.3390/jcm9020469)
Supplement: Supplementary file 1 [file jcm-09-00469-s001.pdf]

Title: Association of rs699947 (-2578 C/A) and rs2010963 (-634 G/C) single nucleotide polymorphisms of the *VEGF* gene, VEGF-A and leptin serum level, and cardiovascular risk in patients with excess body mass: A case–control study.

Authors: Damian Skrypnik, Adrianna Mostowska, Paweł Piotr Jagodziński, Paweł Bogdański

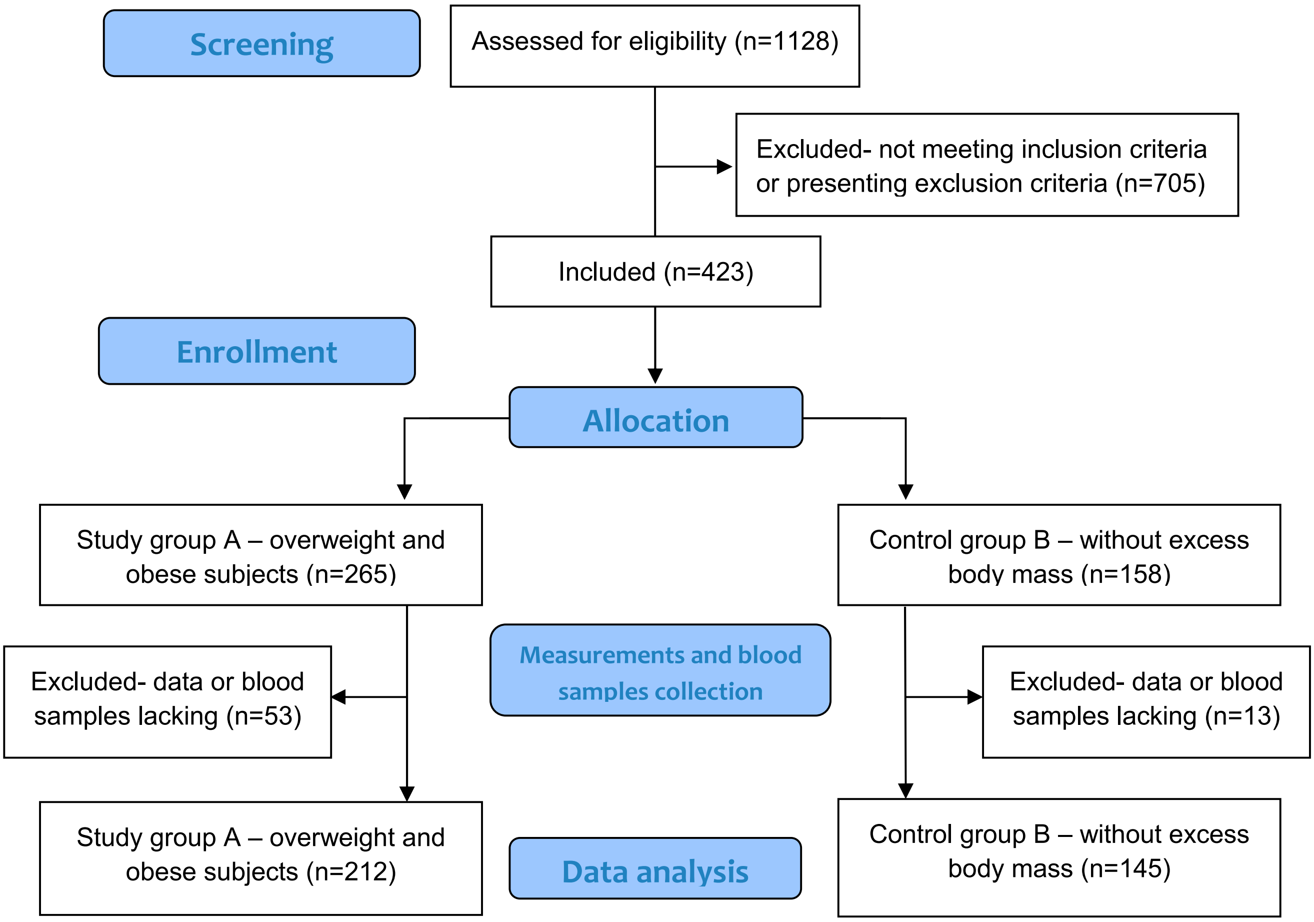

Figure S1. Flow diagram of the study
